# Role of microtubule actin crosslinking factor 1 (MACF1) in bipolar disorder pathophysiology and potential in lithium therapeutic mechanism

**DOI:** 10.1038/s41398-023-02483-6

**Published:** 2023-06-23

**Authors:** Deepak Salem, Ronald J. Fecek

**Affiliations:** 1 Lake Erie College of Osteopathic Medicine at Seton Hill, Department of Microbiology, Greensburg, USA; 2 University of Maryland Medical Center/Sheppard Pratt Psychiatry Residency Program, Baltimore, USA

**Keywords:** Molecular neuroscience, Bipolar disorder

## Abstract

Bipolar affective disorder (BPAD) are life-long disorders that account for significant morbidity in afflicted patients. The etiology of BPAD is complex, combining genetic and environmental factors to increase the risk of disease. Genetic studies have pointed toward cytoskeletal dysfunction as a potential molecular mechanism through which BPAD may arise and have implicated proteins that regulate the cytoskeleton as risk factors. Microtubule actin crosslinking factor 1 (MACF1) is a giant cytoskeletal crosslinking protein that can coordinate the different aspects of the mammalian cytoskeleton with a wide variety of actions. In this review, we seek to highlight the functions of MACF1 in the nervous system and the molecular mechanisms leading to BPAD pathogenesis. We also offer a brief perspective on MACF1 and the role it may be playing in lithium’s mechanism of action in treating BPAD.

## Introduction

Bipolar affective disorder (BPAD) refers to a group of disorders (including Bipolar I, Bipolar II, cyclothymia, and other unspecified bipolar disorders) with a lifetime worldwide prevalence of greater than 2% [1]. Clinically, BPAD consists of recurrent episodes of altered mood, thought process, and emotions that, together, contribute to the significant morbidity associated with these patients [2, 3]. BPAD is highly heritable (up to 93% in a twin cohort in Finland) with many different mutations with small effect sizes combining to increase disease risk [4].

The clinical spectrum, thought to arise from a complex interplay between genetics and environmental factors, can be highly variable. Genome-wide association studies (GWAS) have recently revealed that several genetic risk factors are shared between BPAD, schizophrenia-related disorders, and unipolar depression, suggesting shared underlying mechanisms of disease with the potential for overlapping symptomology [5]. Indeed, BPAD patients typically have alternating episodes of altered mood, including mania and depression, but a significant amount of patients with mania often have concurrent psychotic symptoms [6].

The clinical presentation of patients with BPAD is significant for the optimal choice in pharmacological therapy. Patients with a classical presentation of BPAD, characterized by distinct non-mixed mood episodes, can be remarkably amenable to lithium, known as the gold standard therapeutic in the treatment of BPAD [7]. On the other hand, patients with atypical presentations of BPAD with mixed mood episodes, rapid cycling, and other psychiatric comorbidities may be more resistant to lithium [8]. Other commonly used mood stabilizers, including anticonvulsants and antipsychotics, are efficacious in treating BPAD, but resistance can still occur, highlighting the need for a more thorough understanding of the intrinsic mechanisms behind BPAD to develop new therapeutics [9].

GWAS and next-generation sequencing data suggest molecular mechanisms for BPAD, including (but certainly not limited to) immune-mediated dysfunction, oxidative stress, neurohormonal dysfunction, circadian dysfunction, neurotrophic depletion, cell transport dysfunction, and cytoskeleton dysfunction [10, 11]. Studies examining the molecular mechanism(s) of lithium as a mood stabilizer have revealed several promising targets, including the serine threonine kinase Glycogen synthase kinase 3-β (GSK3B) [12]. Lithium, being a potent inhibitor of GSK3B, has strongly implicated GSK3B and its downstream targets as players in the pathogenesis of BPAD [12, 13]. A downstream target getting more attention is microtubule actin crosslinking factor 1/actin crosslinking factor 7 (MACF1/ACF7), a potent regulator of the cytoskeleton in neurons and glial cells [14]. In this review, we will highlight the growing evidence for the role of MACF1 in BPAD pathology, particularly its ability to modulate the cytoskeleton in neurons. We will also provide a brief perspective on the role MACF1 may be playing in lithium’s mechanism of action in BPAD treatment, and to assist in readability, we have provided a table of abbreviations (Table 1).

**Table 1:** Abbreviations and corresponding descriptions.

Abbreviation	Description
BPAD	Bipolar affective disorder
MACF1/pMACF1/ACF7	(Phosphorylated) microtubule actin crosslinking factor 1/actin crosslinking factor 7
GWAS	Genome-wide association studies
GSK3B	Glycogen synthase kinase 3-β
IF(s)	Intermediate filament
G-actin, F-actin	Globular actin, filamentous actin
Arp 2/3	Actin-related protein complex 2/3
MT(s)	Microtubules
EB	End-binding proteins
DST	Dystonin
BPAG1	Bullous pemphigoid antigen 1
CH	Calponin homology domain
KD, KO	Knockdown, knockout
ATP	Adenosine triphosphate
GAR	Gas2-related domain
MTOC	Microtubule organizing center
EF hand	N-terminal “E” helix, C-terminal “F” helix; A conserved helix-loop-helix
SRD	Spectrin repeat domain
SH3	Src-homology-3 (SH3) protein interaction domain
PRD(s)	Plakin repeat domains
GSR	Glycine serine repeat domain
SNP	Single-nucleotide polymorphism
CNTN6	Contactin, immunoglobulin superfamily protein
CDH13	Cadherin-13
DISC-1	Disrupted in schizophrenia
GABA	γ-Aminobutyric acid
CNS	Central nervous system
NT	Neurotransmitter
MAP1b	Microtubule-associated protein 1b
AChR	Acetylcholine receptor
LTP	Long-term potentiation
AMPAR	α-amino-3-hydroxy-5-methyl-4-isoxazolepropionic acid receptor
CAMSAP3	Calmodulin-regulated spectrin-associated protein family member 3
KIF5A	Kinesin5A
TI-VAMP	Tetanus insensitive vesicle-associated membrane protein
v-SNARE	vesicular Soluble N-ethylmaleimide sensitive factor receptor
TNF-α	Tumor necrosis factor alpha
MIP2/CXCL2	Macrophage inhibitory factor 2/chemokine ligand 2

## MACF1 and the cytoskeleton

### A brief overview of the actin and microtubule cytoskeleton

The cytoskeleton is an incredibly dynamic protein system that is present in essentially all cells [15]. The major elements of the eukaryotic cytoskeleton consist of three main protein filaments: microfilaments made of polymerized actin, microtubules made of cylindrically arranged tubulin monomers, and intermediate filaments (IFs) made from various proteins, such as the keratin of epithelial cells, vimentin from mesenchymal cells, or neurofilaments in neurons [16, 17].

Microfilaments consist of globular actin (G-actin) monomers that are linearly polymerized into filamentous actin (F-actin), which exhibits intrinsic polarity with a plus (growing end) and a minus end (lagging end) [18]. This polarity allows for actin “treadmilling” dynamics, whereby the minus end is depolymerized to allow for the plus end to be elongated further either linearly via the formin family of actin nucleators or elongated in a branching manner via the Arp 2/3 (Actin-related protein) complex [19, 20]. The F-actin cytoskeleton is capable of coupling with the myosin motor proteins, together which can generate force and create a contractile cellular network that can transport subcellular components of many sizes, from synaptic receptors to larger organelles, such as mitochondria [21, 22]. The dynamic nature of actin and its ability to be shaped to the needs of a cell highlight the functional importance of this cytoskeletal element. Indeed, actin is crucial for cell migration, cell division, cell adhesion, signal transduction, and the maintenance of cellular architecture.

Microtubules (MT) are polarized, hollow cylindrical structures made up of heterodimers of α-tubulin and β-tubulin organized from a centrosome, an organelle that can move around in the cytoplasm [23]. Like actin, MTs are incredibly dynamic with two theories of how they grow and shrink: dynamic instability (akin to actin dynamics), where the growing plus end is polymerized and the minus end is depolymerized, and “search and capture” model, whereby the growing end encounters anchoring points which stabilizes further growth at that end [24, 25]. Much akin to actin regulatory proteins, end-binding (EB) proteins can selectively bind the plus end of MTs and enhance growth [26]. Like the myosin motor in actin, MTs can act as substrates for motor proteins such as dynein and kinesin. These proteins essentially “walk” along microtubules in anterograde (kinesin) and retrograde (dynein), carrying protein cargo ranging in size from transcription factors to organelles [27]. MTs play vital roles in cell division, cell migration, cell motility, signal transmission, gene regulation, subcellular trafficking, and in the maintenance of cellular architecture.

These cytoskeletal elements, as dynamic as they are, are not spatially separate. In neurons, dynamic actin networks form the structural basis for the growth and maintenance of dendrites and axons [28]. In dendrite formation and development, microtubules invade the dendrites, and in axons, the centrosome is polarized toward the axon such that microtubules act as a protein trafficking railroad all the way from the more central nucleus to the potentially distant axon terminal [29, 30]. Additionally, neurofilaments are also interwoven spatially between MTs and actin, further regulating axon diameter [31]. As these cytoskeletal systems are so important to the function of cells, many proteins have emerged to interact with each of them individually, and a few protein families have the capability to interact with both simultaneously.

### Structural biology of MACF1

MACF1, as the name implies, is a giant cytoskeletal crosslinking protein that evolved to coordinate these different elements of the cytoskeleton [32]. MACF1 is a member of the spectraplakin family of diverse proteins, with a slightly varying structure to function physiologically in different tissues [33]. The two genes that code for mammalian spectraplakins, *MACF1*, which encodes MACF1/ACF7, and *DST*, which encodes BPAG1 (bullous pemphigoid antigen 1), have several alternative splicing sites allowing for at least 13 different mammalian isoforms with potential yet for further isoform discovery [34].

MACF1 can bind F-actin through two calponin homology (CH) domains that function synergistically: a CH1 domain with a weaker yet capable affinity for actin and a CH2 domain that has a strong affinity for actin [35]. MACF1’s alternative name, ACF7, highlights its importance as an actin-regulating protein. Indeed, knockdown (KD) of MACF1/ACF7 results in disarrayed F-actin networks as well as altered F-actin dynamics in various cell types, including neurons, retinal cells, skin stem cells, osteoblasts, and myocytes [36–40]. Interestingly, MACF1 has an intrinsic ATPase domain that has been shown to interact robustly with F-actin in vitro to achieve ATP hydrolysis levels comparable to picomolar amounts of myosin II and is independent of calcium [38]. This domain was localized to the Walker B motif and was found to be crucial in maintaining proper cytoskeletal dynamics at focal adhesions [38].

The GAR (Gas2-related) domain allows MACF1 to bind MTs with a strong affinity and is capable of binding EB proteins, thus allowing MACF1 to link MTs with other cytoskeletal components [33, 34, 41]. The GAR domain has also been found to stabilize MTs and partially contribute to the resistance to the depolymerizing antineoplastic agent, nocodazole [42]. The GAR domain may potentially regulate MT dynamics via EB protein interactions even when spectraplakins are in a closed confirmation [41, 43]. MACF1 recruitment of EB1 to MT plus tips allows for the linking of itself with other cytoskeletal proteins, such as the actin polymerization protein formin, ultimately permitting further coordinated cytoskeletal movement [44].

MACF1 knockout (KO) in endothelial cells results in longer MTs with skewed trajectories that fail to tether properly to the actin cortex in an in vitro model of wound healing [38]. As these MTs were not able to be anchored to the cortical actin network, longer and more strained MTs resulted [38]. *MACF1* null cultures that were stimulated to elicit wound healing demonstrated impaired polarity of the MTOC (microtubule organizing center) and Golgi, whereby the repositioning of these organelles is pivotal for wound healing and cellular migration [38]. The impairment of cell polarity and altered MT/actin dynamics were rescued with a MACF1 mini gene encoding both the GAR domain and the CH domains, highlighting the importance of MACF1’s ability to bind both actin and MTs in cell migration [38]. Indeed, this same ability of MACF1 to maintain proper cellular polarity during migration is also notable in developing neurons, where MACF1 knockdown resulted in results in destabilized MTs with failed neuronal migration [45].

MACF1 additionally has an EF hand domain (a conserved helix-loop-helix motif found in calcium-binding proteins) that, along with the GAR domain, permits recruitment of EB1 to MT plus tips and consequently link itself with other cytoskeletal proteins such as the actin polymerization protein formin to further orchestrate coordinated cytoskeletal movement [34]. The EF hand domain in spectraplakins is known to bind calcium, implicating MACF1 as a calcium-sequestration protein [42].

There exists a weakly binding MT domain in the plakin domain of MACF1. This plakin domain confers resistance of MTs to colchicine and cold temperature (−20°C), which are both known to promote depolymerization of MTs [46]. This plakin domain has been found in plakin proteins, such as BPAG1e and plectin, to be able to bind B4(β4) integrins [47]. In the case of these plakin proteins, their main roles are to link IFs to cell-extracellular matrix junctions and the membrane to adhesive junctions [47]. With spectraplakins, these plakin domains may be able to bind not only MTs but also integrins, thus linking them through to the actin and MT cytoskeleton.

The spectrin repeat domain (SRD), made of spectrin repeats, links spectraplakins to the larger superfamily of spectrins [33, 34]. These domains are thought to provide spectraplakins with elastic properties to respond to mechanical forces [34, 48]. This domain also includes an Src-homology-3 (SH3) protein interaction domain, which is a structure that can bind several proteins such as the tumor suppressor p53, dynamin, a protein required for synaptic vesicle endocytosis, and CAMSAP3 (Calmodulin-regulated spectrin-associated protein family member 3), an MT-binding protein that is vital for proper neuronal growth [49–51]. The SH3 domain enables spectraplakins to interact with proteins (similar to p53, dynamin, and CAMSAP3) that are involved in cell migration, survival, scaffolding, vesicular transport, and cytoskeleton coordination [48, 49, 51].

Plakin repeat domains (PRDs) are made up of plectin repeats, which structurally are 38 residues forming two antiparallel alpha sheets with a beta hairpin loop [52]. MACF1b is known to have five PRDs [34]. PRDs are able to bind IFs directly, though they potentially act through other mechanisms [52]. Even though plectins lack the typical spectraplakin MT-binding GAR domain and CH actin-binding domain, they have been reported to interact with actin and MTs directly, suggesting that the PRDs’ function is not completely understood [48, 52]. Additionally, the PRD acts as a target to localize MACF1b to the Golgi, and the loss of MACF1b results in altered Golgi complex morphology [53]. The PRDs help to allow spectraplakins to potentially connect the IF, actin, and MT cytoskeletons together, so further study of the function of PRDs would help to illuminate additional roles of MACF1 in undiscovered isoforms.

MACF1 is vital for physiologic development and, as such, is regulated post-translationally via GSK3B, a kinase involved in the Wnt signaling pathway [13, 34]. Through the GSR (glycine serine repeat) domain, GSK3B can phosphorylate MACF1 at six different serine residues, resulting in the conformational change of MACF1 to an “off” position that is unable to bind the cytoskeleton [54]. Overexpression of GSK3B in skin stem cells reduced MACF1 co-localization with MTs, resulting in a dysfunctional MT network similar in phenotype to a MACF1 knockout that is incapable of cytoskeletal coordination [54]. In mouse brain lysate, MACF1 and GSK3B coimmunoprecipitated, and when GSK3B was overexpressed, phosphorylated MACF1 levels (pMACF1) were higher, and neurons were no longer able to migrate properly [55]. These studies demonstrate the ability of GSK3B to directly regulate the function of MACF1.

The functions of MACF1 can be understood through the lens of these domains, though there are further functions that continue to be illuminated for MACF1, such as regulation of the immune system, regulation of mitochondria, protein trafficking, and more that will be discussed later in the context of BPAD.

## MACF1: genetic evidence for role in BPAD and other psychotic disorders

The undeniable contribution of genetic factors to BPAD etiology has more recently resulted in multiple large GWAS studies identifying many single-nucleotide polymorphisms (SNP) that each carries a tiny risk but add up to explain some of the heritability of the disease [56]. The first GWAS to implicate MACF1 as one of these SNP risk factors revealed a spontaneous mutation in the *MACF1* gene of BPAD and schizoaffective patients and emphasized MACF1 as a promising target due to calcium ion binding capability, as sequestration via MACF1 may contribute to alleviating excessive calcium-mediated oxidative damage in neurons [57, 58]. This same GWAS study identified MACF1 as their most intolerant spontaneous mutation, and the Broad Institute gnomAD database assigned MACF1 as having the highest possible intolerance score with strong haploinsufficiency, indicating that due to the theoretical severity in clinical phenotype, highly deleterious mutations in *MACF1* due not persist in the general population genetic pool [57, 59]. The *MACF1* mutation identified in this study by Kataoka et al. (p.V266fs) is a frameshift mutation that is predicted to result in nonsense-mediated mRNA decay and, ultimately, decreased MACF1 protein synthesis [57, 60].

Two other studies have since validated *MACF1* as an important risk gene in BPAD: A whole exome sequencing (WES) study in a cohort of 81 patients from 27 different families was able to again report *MACF1* as a significantly enriched mutation, and another separate cohort of 53 BPAD patients found 10 cases of non-silent *MACF1* mutations [61, 62]. More recently, this same frameshift mutation (p.V266fs) was knocked in using CRISPR/cas9 in mice, which found that mice with this loss of function mutation in MACF1 experienced behavior consistent with BPAD, such as impaired reward processing and lower attention ability [60]. Another recent mouse study with *Macf1*-deficient mice resulted in social deficits and anxiety-like behavior [36]. These mice model studies of MACF1 deficiency strengthen the association between MACF1 and BPAD.

A *MACF1* missense mutation (p.T4642S) that co-segregated with duplicate variants in CNTN6 (contactin, immunoglobulin superfamily protein) and CDH13 (cadherin-13) was associated with a rare familial psychosis with the family having genetically affected members with schizophrenia and schizoaffective disorder—two psychiatric illnesses with significant genetic overlap with BPAD [63, 64]. Interestingly, in this family, the MACF1 missense mutation (p.T4642S) was in the spectrin repeat domain—a part of MACF1 that interacts heavily with other proteins [63]. Additionally, MACF1 was found to be hyper-methylated in a cohort of patients with first-episode psychosis with presumed schizophrenia diagnoses [65]. The overlapping risk of BPAD and schizophrenia with MACF1 has also been established through other variants found in schizophrenia and through the interactions between MACF1 and DISC-1 (Disrupted in Schizophrenia) [66]. Mutations in DISC-1 are known risk factors for both schizophrenia and BPAD [67]. DISC-1 has been demonstrated to function similarly to lithium, being able to also inhibit GSK3B and further highlighting its potential function in BPAD [68]. Indeed, the functions and hypothesized interactions of DISC-1 in regulating the cytoskeleton and cellular trafficking are thought to be mediated by MACF1, further stressing the importance MACF1 plays in these diseases [66].

## Role of MACF1 in BPAD etiology

The proposed pathophysiological mechanisms of disease in BPAD include intrinsic genetic/epigenetic risk interfacing with environmental stimuli resulting in alterations in the biology of the nervous system. Some of these factors can be reversible, such as the phosphorylation of key proteins like GSK3B, and others can be insidiously progressive, as is the long-term loss of cortical gray matter in BPAD patients. In this section, we will discuss the role MACF1 plays in some of the proposed mechanisms of BPAD etiology and neuroprogression.

### MACF1 in nervous system development

One mechanism of BPAD risk and etiology is dysfunctional neuronal migration in the development of the central nervous system (CNS). Physiological brain development requires precise migration patterns and cellular positioning [69]. Inhibitory GABAergic interneurons migrate tangentially from the ventral telencephalon and then change position and direction to migrate radially to their final location in the neocortex [70, 71]. Excitatory neurons, contrastingly, migrate radially [72]. In BPAD, there are altered ratios of excitatory neurons and inhibitory interneurons in the prefrontal cortex and hippocampus, possibly due to defective neuronal cell migration and increased neuronal apoptosis [73–76].

The coordination and orientation of actin and MTs are crucial here, as polarity defects of these components result in altered cellular migration. This need for cytoskeletal organization is highlighted by the strong CNS expression of MACF1 overall and during development [77, 78]. In neuron-specific *Macf1* deletions in mice, migration of excitatory pyramidal neurons and in inhibitory GABAergic interneurons were suppressed with aberrant positioning of neurons along their migratory paths [45, 79]. Radially migrating neurons destined to be functional excitatory pyramidal cells had destabilized MTs and static, rather than dynamic centrosomes that altered their migratory potential [79]. Radial neuron progenitors have altered F-actin distributions and destabilized MT populations in *Macf1* CNS conditional knockout mice and together with the cellular heterotopia demonstrated in the cortical layers of *Macf1*-deficient mice, support the role of MACF1 in not only radial cell migration but perhaps neurogenesis [14, 36, 78]. The complex journey of GABAergic interneurons is similarly perturbed in neuron-specific *Macf1* KO, with these interneurons phenotypically expressing disrupted MT stability, uncoordinated migrating leading edges, slower migration speeds, and an inability to transition from tangential to radial migration [75].

Notably, in the CNS, the ability of MACF1 to coordinate the cytoskeleton for neuronal migration is regulated by GSK3B [14]. Constitutively active GSK3B leads to increased pMACF1 (inactive MACF1) and subsequent aberrant neuronal migration [79].

### MACF1 in axon and dendrite function

Neuronal circuits consisting of properly connected dendrites and axons serve as the basis for the physiological function of the brain [80]. These axons and dendrites can serve as highly active biochemical sites, where ionic exchange due to input can trigger local protein signaling, resulting in a functional output such as neurotransmitter (NT) release [80]. Dynamic cytoskeletal coordination is key in proper dendritic spine architecture and morphogenesis, both of which are intrinsically tied to the functional ability of synapses [81]. Although most of the excitatory synapses occur at dendritic spines, a single spine can receive both inhibitory and excitatory inputs, stressing the importance of the proper length and architecture of a spine in the grand scheme of the neuronal circuitry inside the brain [82].

BPAD is associated with decreased dendrite length, density, and branching—all of which are significant to proper plasticity of synapses [82, 83]. Indeed, BPAD is associated with altered structural plasticity of these dendritic spines and BPAD has been associated with altered excitatory/inhibitory synapse ratios [82, 84, 85]. All these mechanisms point toward another hypothesis in BPAD etiology: altered morphological function in dendritic spines and axons.

Selective MACF1 KO in mice cortical neurons results in decreased length, density, and branching of dendritic spines [55]. These dendritic spines are thinner with significantly decreased spine head and neck size [55]. Contrastingly, the control neurons had several spines with more typical mushroom-like dendritic spine phenotypes [55]. This stark difference is notable particularly as mushroom-like dendritic spines are considered functionally active, with NT receptors present at the interface of a synapse with another neuron [86]. In MACF1-depleted neurons in zebrafish and in mice, the actin and MT cytoskeletons are perturbed [36, 45, 55, 79, 87]. In the altered dendritic spines of MACF1-depleted neurons, polymerized F-actin were scattered, de-bundled, and concentrated closer to the soma of the neuron rather than at the tip of the supposedly growing spine [55]. Additionally, in MACF1 KO neurons, MTs were also de-bundled and mislocalized away from the spine tips, further suggesting a separation of the actin and MT cytoskeleton systems not only spatially, but functionally [55]. The role that MACF1 plays in maintaining dendritic spine morphology is similarly present with axons. Decreased axonal length and branching were found in MACF1-deleted mouse callosal neurons [55]. Similarly, in a human family, mutations in the MT-binding GAR domain of MACF1 similarly caused defects in axon extension [88]. MACF1 additionally binds and acts as an anchor for MAP1b (microtubule-associated protein 1b), a protein of significance in axonal elongation and dendritic spine morphology [40]. MACF1 recruits the MAP1b/ EB1/Vinculin/beta-tubulin complex to stabilize acetylcholine receptors (AChR) at the postsynaptic membrane through Rapsyn and F-actin [40]. A loss of MACF1 decreases AChRs at the membrane due to destabilization because of the absence of this complex, demonstrating another mechanism through which MACF1 can affect synapse function [40].

Proper cell transport along both polymerized actin and MT cytoskeletons is necessary for synapse function and long-term potentiation (LTP), a form of structural Hebbian plasticity [81, 85, 89, 90]. LTP is associated with significant changes in the numbers, size, and stability in dendritic spines, further cytoskeletal rearrangements, and increased NT receptor localization of AMPAR (α-amino-3-hydroxy-5-methyl-4-isoxazolepropionic acid receptor) to the synapse [91]. Perturbed LTP and structural plasticity is seen in BPAD [85, 92, 93].

MACF1 has direct and indirect roles in regulating actin and MT cytoskeleton-mediated transport in neurite growth. MACF1, through directly affecting MT stability and F-actin morphology, may affect the ability of cytoskeletal protein motors like kinesin, dynein, and myosin motors to transport cargo to synapses [94, 95]. MACF1, through its SRD, can bind CAMSAP3 (calmodulin-regulated spectrin-associated protein family member 3), a MT minus end protein important in MT stability [50]. CAMSAP3 is an essential protein in neuronal extension and dendrite growth, providing yet another mechanism for MACF1 to regulate transport [51]. Additionally, MACF1 plays a role in protein transport directly via interactions with the Golgi complex directly (via the PRD domain) and indirectly through its interaction with p230/GolginA4 [53, 96]. MACF1 acts as a molecular link between GolginA4 and the MT cytoskeleton to bind KIF5A (kinesin5A) to allow TI-VAMP (tetanus insensitive vesicle-associated membrane protein) v-SNARE (Soluble N-Ethylmaleimide Sensitive Factor Receptor) tagged vesicles to reach growing neurite tips [97]. This process mediated by KIF5A is necessary for axonal growth and the maintenance of dendrite morphology [98–100]. The recruitment of EB1 through EF hand domains and subsequently MAP1b is pivotal for a healthy synapse as MAP1b regulates MT stability and transport [89, 101]. Indeed, loss of MAP1b significantly impairs hippocampal LTP, providing yet another outlet that MACF1 regulates synapses [89].

### MACF1 as an immune system modulator

Accumulating evidence implicates immune system dysfunction in BPAD etiology. BPAD is associated with increased levels of pro-inflammatory cytokines and dysfunctions of the adaptive and innate immune system [102–104]. Microglia, which are the resident tissue macrophages of the CNS, are known to play a role in the pathogenesis of BPAD [105]. Further evidence for a dysregulated immune system in BPAD stems from lithium’s ability to act as a potent anti-inflammatory agent, decreasing upregulated levels of IL-4, IL-6, IL-10, and TNF-α in a mouse model of mania [106].

In mouse RAW264.67 macrophages, *Macf1* deletion resulted in increased levels of IL-6 [107]. In a mouse model of colitis, MACF1 KO resulted in increased TNF-α and increased MIP2/CXCL2 (macrophage inflammatory protein 2) [108]. MACF1 facilitates the nuclear translocation of SMAD7, a significant and potent anti-inflammatory protein [109, 110]. Additionally, MACF1 is upregulated in helper T cells following GSK3B inhibition and functions potentially to facilitate cytokine secretion at the immunological synapse [111]. These studies demonstrate that MACF1 plays a role in regulating the innate immune system and the adaptive immune system as well as potentially acting as an immunosuppressor, that when decreased in levels, may increase the risk of BPAD.

### MACF1 and mitochondrial function

Mitochondria function to regulate a key number of cellular processes, including bioenergetics, metabolism of free radicals, autophagy, and calcium homeostasis [112]. Significant evidence points toward mitochondrial dysfunction as an etiology in BPAD pathogenesis [112]. Mitochondrial dysfunction can worsen pro-inflammatory states, dysregulate calcium homeostasis, and potentially damage neurons through the accumulation of free radicals [112, 113].

Morphologically in BPAD, mitochondria are misshapen and functionally altered [114]. In the absence of MACF1 in mouse myocytes, the morphology of mitochondria is perturbed [40, 115]. Additionally, *Macf1* deletion in mice myocytes also resulted in altered bioenergetics and altered levels of proteins involved in mitochondrial biogenesis [115]. MACF1, through the EF domain, functions to sequester calcium and in doing so, can prevent mitochondria from further calcium toxicity-induced free radical production.[42, 116].

## Perspective on MACF1 and lithium

Even though lithium is considered the gold standard in treating classical BPAD, its exact mechanism of action is not completely understood [117]. Lithium’s potent indirect and direct inhibition of GSK3B is widely considered as playing a significant role in the drug’s mechanism and therapeutic efficacy in BPAD [12]. GSK3B is a strong player in BPAD etiology, with levels of the enzyme being elevated during manic episodes in patients with BPAD and mouse studies of GSK3B perturbation demonstrate mania-like behaviors [118]. Both GSK3B and lithium are known to be important in normal synaptic plasticity, neuronal migration, immunomodulation, and cell transport—just as MACF1 plays a role in these same processes [14, 107, 117, 118]. GSK3B is a known endogenous inhibitor of MACF1 via phosphorylation, forcing pMACF1 to an “off” conformation that is unable to bind the various components of the cytoskeleton [14, 54, 55]. Additionally, it has been well established that GSK3B inhibition, via lithium (and other direct GSK3B inhibitors), raises unphosphorylated “on” MACF1 levels [54, 119, 120].

**Fig. 1:**
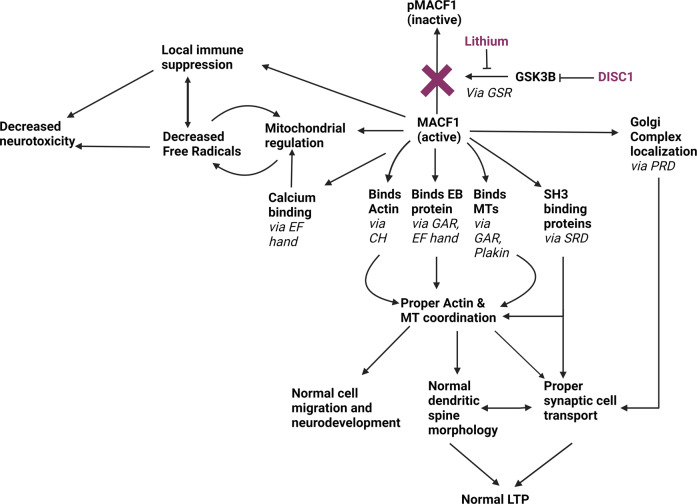
Proposed functions of MACF1 in the therapeutic pathway of lithium in BPAD. Lithium (and DISC1 endogenously) inhibits GSK3B, raising levels of active MACF1. Active MACF1 then functions (through domains added in italics) in neurons to re-normalize pathologically altered neuronal structure and function in treating BPAD symptomology. MACF1 microtubule actin crosslinking factor 1, pMACF1 phosphorylated microtubule actin crosslinking factor 1, BPAD bipolar affective disorder, DISC1 disrupted in schizophrenia 1, GSK3B glycogen synthase kinase 3β, MT microtubule, CH calponin homology, GAR Gas2-related, SH3 Src-homology-3, SRD spectrin repeat domain, PRd plakin repeat domain, LTP long-term potentiation. Created with Biorender.com.

We hypothesize that in patients with BPAD with elevated GSK3B levels, active MACF1 levels will be lower, resulting in altered neurobiology and progression of disease process. Additionally, we hypothesize that lithium, through its inhibition of GSK3B, raises active MACF1 levels to therapeutic levels and ameliorates at least some of the altered neurobiology in BPAD. Patients with BPAD and MACF1 complete loss of function (LoF) mutations may have clinical resistance to lithium, as lithium, working upstream, would not be able to restore function in this case. We propose that lithium, through MACF1, acts to normalize altered synaptic functions of neurons, aberrant dendritic spine morphology, dysfunctional cytoskeleton-based cell transport, dysregulated immune system, and dysfunctional mitochondria (Fig. 1).

## Future directions

The precise pathogenesis of BPAD is still continuing to be illuminated. Additionally, MACF1 is clearly a complex protein with many different functions. The role MACF1 plays in BPAD through genetics and molecular interactions will continue to be discovered, just as the therapeutic mechanism of lithium will continue to be elucidated. Future studies could explore the range of known effective mood stabilizers including those acting via sodium channels, such as carbamazepine, valproic acid, and lamotrigine or antipsychotics to identify any contributions from our proposed GSK3B/MACF1 mechanisms. Our hypothesis can also be validated in emerging GSK3B inhibitors as they are trialed in BPAD [121, 122]. Investigation into various mutational polymorphisms in lithium resistance will also be elucidative and potentially of clinical predictive value. Given its central role across neuropathological mechanisms in BPAD, MACF1 could potentially be useful as a biomarker in the diagnosis and prediction of treatment response. Further analysis of its biological function will improve our understanding of these relationships.
